# RF system calibration for global Q matrix determination

**DOI:** 10.1016/j.mri.2015.12.039

**Published:** 2016-06

**Authors:** Francesco Padormo, Arian Beqiri, Shaihan J. Malik, Joseph V. Hajnal

**Affiliations:** a King's College London, Division of Imaging Sciences and Biomedical Engineering, The Rayne Institute, 3rd Floor, Lambeth Wing, St Thomas' Hospital, London, UK, SE1 7EH; b King's College London, Centre for the Developing Brain, Division of Imaging Sciences and Biomedical Engineering, The Rayne Institute, 3rd Floor, Lambeth Wing, St Thomas' Hospital, London, SE1 7EH, UK

**Keywords:** Parallel transmission, SAR, MRI Calibration, Q Matrix

## Abstract

The use of multiple transmission channels (known as Parallel Transmission, or PTx) provides increased control of the MRI signal formation process. This extra flexibility comes at a cost of uncertainty of the power deposited in the patient under examination: the electric fields produced by each transmitter can interfere in such a way to lead to excessively high heating. Although it is not possible to determine local heating, the global Q matrix (which allows the whole-body Specific Absorption Rate (SAR) to be known for any PTx pulse) can be measured in-situ by monitoring the power incident upon and reflected by each transmit element during transmission. Recent observations have shown that measured global Q matrices can be corrupted by losses between the coil array and location of power measurement. In this work we demonstrate that these losses can be accounted for, allowing accurate global Q matrix measurement independent of the location of the power measurement devices.

## 1 Introduction

Parallel transmission (PTx) is becoming ever more widely utilized in MRI systems operating at or above 3 tesla (T) to overcome radiofrequency (RF) transmit field (B1 +) inhomogeneity and accelerate RF pulses [1], [2], [3]. A remaining open question is how to guarantee patient safety when using PTx technology. During RF transmission, the electric fields produced by each transmit element interfere, potentially leading to constructive superposition and consequently patient heating [4] above regulatory local and whole-body specific absorption rate (SAR) limits [5].

Two primary approaches have been developed to infer electric field and heating information of transmit elements for local SAR estimation. The first involves solving Maxwell's equations numerically using finite-difference time-domain (FDTD) techniques for a digital model of the patient and coil system, obtaining the RF magnetic and electric fields across the entirety of the subject [6]. However, this approach has limited applicability as the digital models are not currently patient-specific and do not account for posture dependent variations. The second approach involves the inference of electric field and patient heating information directly from MRI measurements [7], [8], [9]. Whilst these burgeoning methods provide patient and situation-specific data, they are still in their infancy and normally incur a significant time penalty.

In contrast to local SAR estimation, a method enabling patient-specific measurement and prediction of global SAR for arbitrary PTx pulses was recently presented [10]. This is achieved by equipping the MRI RF system with power meters (PMs) and a directional coupler (DC) for each transmit channel. By monitoring the RF power delivered to and reflected by each transmit element whilst transmitting on multiple different linear combinations of channels, it is possible to determine the global Q matrix [11], which describes the volume-integrated interferences of electric fields from all transmitters. The global Q matrix enables the prediction of RF power deposition in the MRI system, and can be used as a constraint in PTx pulse design calculations [12], [13].

It has recently been noted that measured global Q matrices (**Q**_meas_) do not solely contain the desired information regarding the electric fields present in patients (**Q**_pat_), but are also contaminated by other power loss mechanisms (**Q**_other_) [10], as described by Eq. (1). These additional losses make any SAR predictions made using **Q**_meas_ inherently an over-estimate. Whilst it is favorable to make conservative SAR estimates, it would be preferential to characterize **Q**_other_ so that **Q**_pat_ is known as accurately as possible.

(1) $$Q_{\mathrm{means}}=Q_{\mathrm{pat}}+Q_{\mathrm{other}}$$

A recent paper analyzed many of the additional loss mechanisms: power is deposited in the physical coil (i.e. losses in metal, substrate and lumped elements, denoted by **Q**_coilHW_) and electromagnetically radiated (**Q**_rad_) [14]. Although these losses can be analyzed through FDTD simulations as demonstrated in the cited paper, these mechanisms are unfortunately very difficult to quantify in practical scenarios. A further important loss mechanism has also recently been highlighted [13]: losses in coaxial cables located between DCs and the transmit array can severely impact global Q matrix measurement. Significant cable losses are often experienced when it is not feasible to have the DCs directly adjacent to the transmit array, either due to cost-limitations or the inability of these devices to operate in a magnetic field. In this work we demonstrate a simple correction method to account for these cable losses, resulting in more relevant, patient-specific power deposition predictions independent of DC placement along the RF transmit chain.

## 2 Theory and methods

### 2.1 Theory

**Fig. 1:**
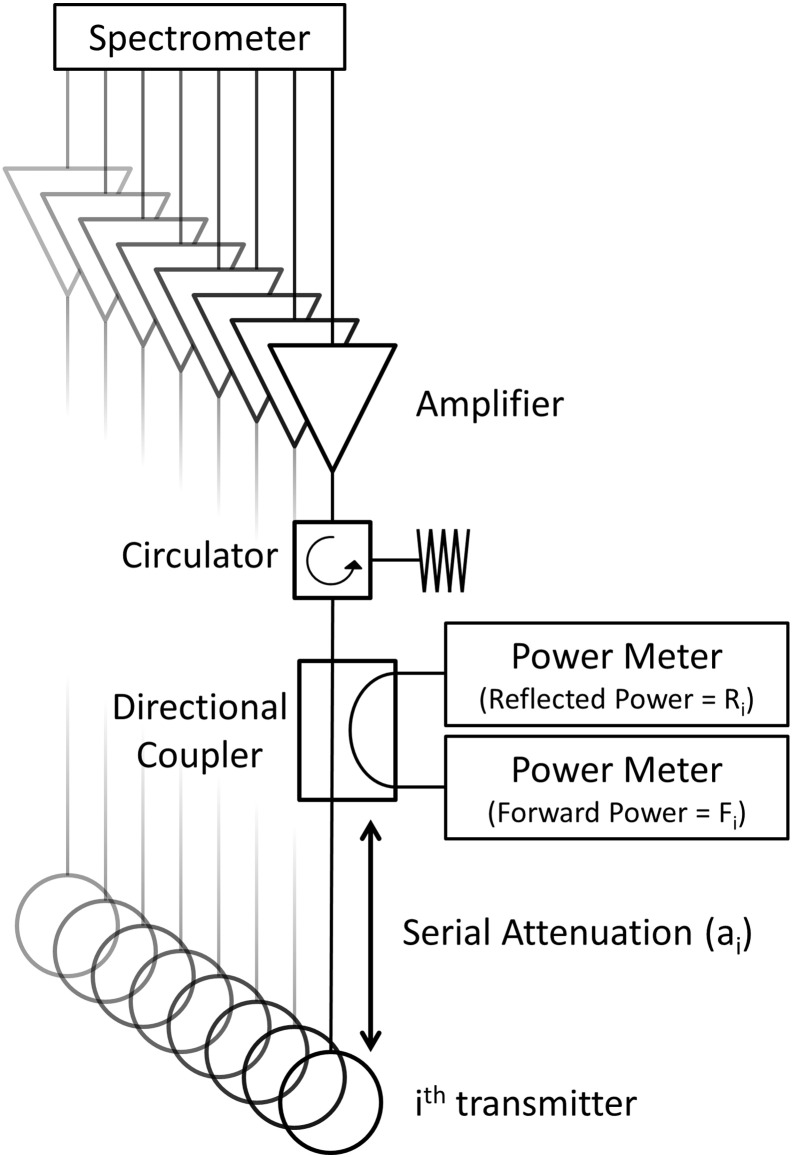
Example transmit system equipped with directional couplers and power meters to enable global Q matrix measurement.

Consider the MRI system equipped with N_T_ transmit channels shown in Fig. 1. A single spectrometer generates N_T_ low-level RF pulses, which are subsequently amplified and passed to a transmit array via circulators and loads which ensure all components are protected from reflections. Each transmit channel is also equipped with a DC, which directs a small fraction of the incident forward and reflected voltage waves to PMs for measurement. The RF path between the i^th^ DC and transmitter can be described by a single complex parameter a_i_, which describes the signal attenuation and phase accrual as an electromagnetic wave that travels from the DC to the transmit element.

Global Q matrices are determined by performing a series of N_M_ measurements. Each measurement involves continuous RF transmission, with the applied amplitudes and phases of each transmit channel, referred to as RF shims, denoted by the N_T_x1 complex vector **w**_j_ (j = 1, 2,..., N_M_). The PMs measure the power of the electromagnetic waves traveling towards (F_i,j_) and away (R_i,j_) from the transmit array at the location of the DC concurrent with transmission. By invoking the principle of conservation of energy, the total power absorbed by the system, d_j_, is given by Eq. (2).

(2) $$d_{j}=\sum_{i=1}^{N_{T}}F_{i,j}-R_{i,j}=w_{j}^{H}Q_{\mathrm{means}}w_{j}$$

All power difference measurements can be concatenated into the N_m_x1 vector **d**; a ‘weights’ matrix **W** contains all of the applied RF shims, and the vector **q**_meas_ contains all of the individual Q matrix elements, which are obtained by calculating **q**_meas_ = **W**^− 1^**d**.

In order to eliminate the influence of cable losses, the appropriate terms in Eq. (2) must be corrected using knowledge of the cable attenuation. This is achieved by undergoing a process referred to as ‘shifting the reference plane’ [15], which shifts the effective point of measurement (i.e. the location of the DCs) from their remote location to directly adjacent to each transmit element, as demonstrated by Eqs. (3a), (3b).

(3a) $$d_{j}^{\mathrm{corr}}=\sum_{i=1}^{N_{T}}F_{i,j}^{\mathrm{corr}}-R_{i,j}^{\mathrm{corr}}=\sum_{i=1}^{N_{T}}{\left|a_{i}\right|}^{2}F_{i,j}-R_{i,j}/{\left|a_{i}\right|}^{2}$$

(3b) $$d_{j}^{\mathrm{corr}}=w_{j}^{\mathrm{cor}r^{H}}Q_{\mathrm{corr}}w_{j}^{\mathrm{corr}}$$

Eq. (3a) describes the power deposited during transmission directly at the ports of the transmitters, d^corr^. The power difference is described by the forward and reflected powers at the coils, given by F^corr^ and R^corr^, respectively. These quantities can be obtained directly from the recorded forward/reflected powers by multiplying/dividing them by the square of the attenuation factor. This can be understood as follows: the forward voltage wave amplitude at the DC (V_DC_) reduces by factor *a* en-route to the transmitter (V_coil_ = aV_DC_); as the power is proportional to the square of the voltage, the measured forward power needs to be reduced by factor a^2^. Conversely, the measured R_i,j_ is too small as the reflected voltage wave has been attenuated en-route back to the DC for sampling, and therefore needs to be corrected by dividing by a^2^.

Eq. (3b) describes the corrected quadratic term where w_j,i_^corr^ = a_i_w_j,i_; this reflects the fact that the driving voltages at the coil ports are attenuated beyond the DCs.

The process of determining **Q**_corr_ follows the original process. The vector **d**^corr^ is formed from the corrected power readings, and the matrix **W**^corr^ is constructed using the modified RF shims.

## 3 Methods

The proposed correction method was tested experimentally using a 3 T MRI system with a 4 channel transmit array consisting of rectangular loops mounted on a cylindrical former (PulseTeq Ltd., Surrey, UK); adjacent coils were decoupled geometrically, with no further decoupling measures utilized for opposite elements. Experiments were performed using a Philips Achieva console modified for parallel transmission driving Analogic AN8134 RF power amplifiers. Directional couplers (Werlatone C8904, 50 dB coupling factor) were placed at the scanner faraday cage penetration panel. The transmit elements were each connected to the penetration panel by 1.3 m of RG223/U low-loss coaxial cable (estimated cable losses < 0.19 dB), with additional switched attenuators (Pasternack PE7036-1) placed in-line with the transmit elements. The forward and reflected ports of the DCs were connected to a Pickering multiplexer (40-874-002 mounted in National Instruments PXI-0133 chassis), and then to two Rohde & Schwarz NRP-Z11 power meters. The multiplexer and PMs were controlled in Matlab (The Mathworks) and synchronized with the console using the RF amplifier TTL output.

The RF shims used for the measurements were designed as prescribed by Zhu [10]. Sixteen measurements were performed for Q matrix determination, followed by a set of forty-eight random RF shims to test the predictive capabilities of the measured Q matrices. All of the measurements were measured sequentially in a single RF pulse, with each shim being transmitted for 0.61 ms. The RF pulse was repeated four times; for each, the two PMs would be connected to the two ports on a single DC, with switching to different DCs using the multiplexer for subsequent measurements.

The coil array was tested with two different configurations; loaded asymmetrically with a 2 L phantom containing saline (conductivity = 18 S.m^− 1^). For each configuration, two experiments were performed. First, total forward and reflected power was measured to determine the true gold standard Q matrix of the coil, Q_GS_. Secondly, 3 dB of attenuation was switched in on all channels to provide voltage wave attenuation between the directional couplers and the coil elements to simulate increased cable losses.

The second dataset was processed twice. The matrix Q_NC_ (NC = no correction) was calculated by following the methodology as proposed by Zhu; Q_FC_ (FC = full correction) was obtained by following the proposed methodology.

The two matrices were then used to predict the power deposited for the random RF shims and compared for accuracy to the measured power differences obtained without any in-line attenuation.

## 4 Results and discussion

**Fig. 2:**
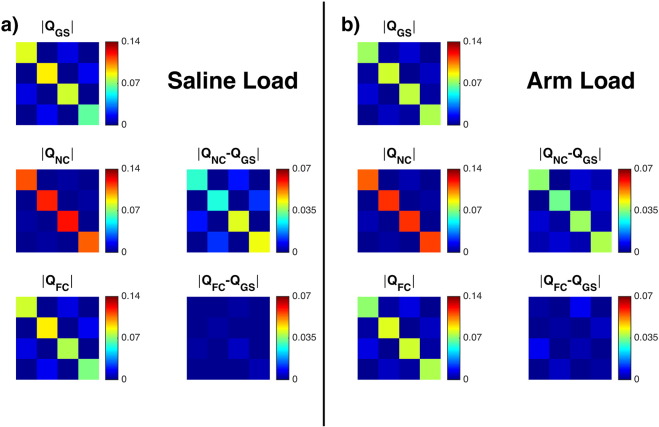
Reconstructed Q matrices (left column of each sub-figure) and their error with respect to the gold standard (right column); a) coil array loaded with saline, and b) coil array loaded with arm. The units of each Q matrix element is W/(unit drive)^2^.

Fig. 2a displays the results with the transmit array loaded with saline. The left hand column shows the gold standard Q matrix, Q_GS_, obtained with no serial losses present. The diagonal elements have varying amplitudes (as the load was positioned asymmetrically), and the off-diagonal elements corresponding to non-decoupled opposite elements are non-zero. The matrix Q_NC_ is obtained with 3 dB serial attenuation on all channels. It is significantly more diagonal than the original, as previously observed [13], and the off-diagonal elements are attenuated. Utilizing the correction method proposed in this paper, a Q matrix with only small differences with respect to the original is obtained. Very similar results are obtained with the coil loaded with a human arm, as seen in Fig. 2b.

**Fig. 3:**
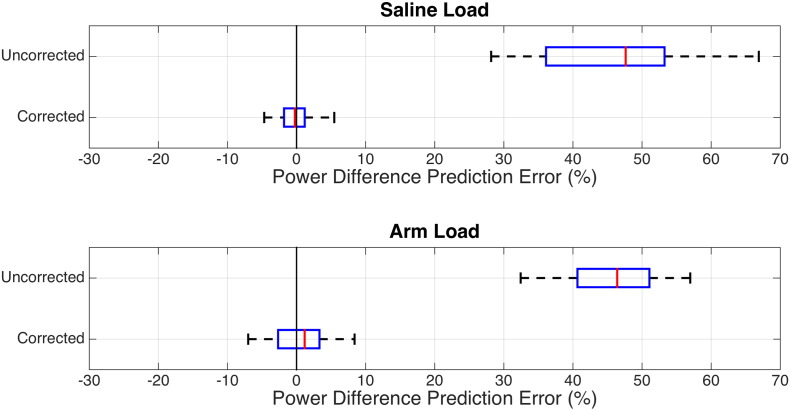
Box plots showing the accuracy of power predictions using both uncorrected and corrected Q matrices versus measured power differences without in-line attenuation. The dashed lines indicate the extremal values; the blue boxes show the 25th and 75th percentile, and the red bar indicates the median.

Fig. 3 shows the results of comparing the *predicted* power deposition using both the uncorrected and corrected Q matrices to the *measured* power deposition without any in-line attenuation. The box plots in fig. 3 summarize the fractional error for each of the 48 random RF shims. The predicted power using the corrected Q matrix produces predictions which reflect the actual power deposition in the load; the uncorrected Q matrix error reflects the fact that it contains significant information about losses between the DCs and the transmit array. The proposed corrections are successful for both the array loaded with saline and with a human forearm.

The method proposed in this paper requires knowledge of the coaxial cables transmission coefficients. These can be obtained by a simple calibration procedure at scanner installation, and would not be expected to change thereafter.

This paper has demonstrated that accounting for losses between the DCs and transmit array allows for measured Q matrices to reflect closer the losses in the transmit array itself. This method cannot account for the various sources of loss beyond the ports of the array; the balance between subject losses and hardware/radiative losses will depend on the subject and design of the coil array [14].
